# Steering diversity — cellular aspects of gene flow

**DOI:** 10.1007/s00709-024-01934-1

**Published:** 2024-02-08

**Authors:** Peter Nick

**Affiliations:** Joseph Gottlieb Kölreuter Institute Für Plant Sciences, Karlsruhe Institute of Technology, Karlsruhe, Germany

The evolution of sexuality has inspired numerous questions ever since. While Woody Allen’s iconic movie “Everything You Always Wanted to Know About Sex But Were Afraid to Ask” is mainly addressing social taboos around sexuality, even biologists have to deal with numerous questions that have remained enigmatic. This begins already with the cell biology of this fundamental process. It appears straightforward that meiosis must have derived from mitosis. However, the combination of chromosome pairing, recombination between chromatids of the two parents, suppressed separation of sister chromatids during first metaphase, and the omission of DNA synthesis during the second division is extremely complex. How such a complex trait might have arisen seems enigmatic and difficult to understand. An interesting hypothesis (Wilkins and Holliday 2009) proposed that pairing originally had the function to suppress, rather than to enable recombination between parental chromosomes. Even without this recombination, the merging of two chromosomal sets and their subsequent redistribution to the gametes would lead to considerable variation — the raw material fuelling Darwinian evolution. For a number of n chromosomes, there are 2^n^ possibilities to assign them to gametes. The second driver of variation — intrachromosomal recombination — might have been added in a later step. Mitotic gene flow is strict — from the mother cell to their daughters. Meiotic gene flow allows diversions and ramifications culminating in genetic diversity. Under favourable conditions, mitosis is more efficient, because it generates more offspring per time. Things become different, when an organism is facing a challenging environment, where change is needed to allow for adaptation to the challenge. Thus, sexuality is a form of gene flow that is advantageous under specific circumstances. Therefore, the obligate sexuality found in many metazoan animals seems to be a specialised case. Originally, sexuality was facultative, which means that organisms must have ways to control gene flow and, by this way, also to steer their diversity. Three contributions to the current issue reflect different aspects of this fundamental cell biological capacity.

The progress in phylogenomics beyond the usual model species has allowed novel insights into the crucial transition from water to a terrestrial lifestyle. This transition came with innovations in the field of sexuality. The contribution by Kurtović et al. (2024) reviews the re-newed interest into one of these transitional forms, the Green Alga family *Characeae*. These highly complex algae have been a classic model of cell biology over decades. For instance, the analysis of plasma streaming in *Nitella* led to the discovery of non-muscle actomyosin (Kamiya and Kuroda 1956), the heavy *Glanzkörperchen* in the rhizoid were central models for the analysis of gravity sensing (for review, see Braun and Limbach 2006), and the giant internodal cells were ideal models for electrophysiology, enabling the characterisation of novel ion transporters (Quade et al. 2022). Since these Streptophyte algae had been recalcitrant to molecular approaches, they fell out of fashion outside the cell biological community. This has strongly changed during recent years, mainly due to the establishment of a high-quality genome that revealed numerous innovations considered as “pre-adaptations” for a terrestrial lifestyle. Contrasting with most Chlorophyceae, the Characeae are endowed with true sexuality, albeit asexual propagation occurs as well. Their elaborate sexual organs occur side by side in the later nodes, and fertilisation leads to a so-called oospore (actually a zygote) that is able to remain dormant and to persist even harsh conditions. The morphology implies self-fertilisation, which somewhat reduces the variability enabled by the sexual process. However, the antheridia mature earlier, such that cross-fertilisation is favoured. Some species of *Chara* are even diecious, which enforces obligate outcrossing. The finding that sexual propagation is not obligate but dependent on light intensity (Sato et al. 2014) shows that plants like *Chara* have acquired the ability to steer their gene flow already prior to terrestrialisation. Signal-regulated sexuality might have been one of the key innovations enabling the step from water to solid land.

The end point of this terrestrial evolution is represented by orchids that use elaborate mechanisms to ensure gene flow within the borders of the species. The contribution by Ricci et al. (2024) describes an additional genetic barrier, for the first time of the important group of the New World Maxillariinae orchids. They provide clear anatomical and cellular evidence for gametophytic self incompatibility, where pollen tubes deriving from the same plant are recognised as self and aborted on the way by a specific interaction with the stylar tissue. Thus, gene flow is under much stricter control as in *Chara*, which renders these anyway endangered plants even more vulnerable, because they cannot bridge the lack of mating partners by self-pollination. Here, the tight control of gene flow might have reached a state, where it exerts already negative effects on fitness.

Also, the contribution by da Silva and Costa-Leonardo (2024) deals with cellular mechanisms regulating fertilisation, here in eusocial insects, termites. The evolution of cooperative behaviour is intimately linked with a high degree of genetic relationship, such that the fitness benefit of social behaviour exceeds that of selfishness. In the Hymenoptera, kinship is established by a brief copulation period of the queen combined with male haploidy. From a genetic point of view, bee drones can be considered as the flying sperm of the queen. Sperm storage in spermatheca is, therefore, a crucial factor for eusociality. The evolution of eusociality in termites is a convergent phenomenon, and, different from the Hymenoptera, it is achieved by life-long copulation between a royal couple. Nevertheless, termites do possess spermatotheca, but their function is not well understood. Using the New-World termite *Cryptotermes brevis* as paradigm, authors follow development, secretion and histochemistry of spermatheca and colleterial glands. Authors show that mating strongly enhances secretion of polysaccharides and proteins in the spermatheca, enabled by ultrastructural changes related to secretion. Conversely, the colleterial glands become active in the context of oviposition. In other insects, colleterial secretion is linked with egg coatings. However, termites do not produce those. Here, the function might rather be lubrication of genital ducts for smoother oviposition. The ultrastructural changes are dependent on development and mating competence. Since the sperm lacks flagella and, in consequence, is not motile (Laranjo et al. 2020), it is these cellular aspects of the female organs that decide on fertilisation and, thus, gene flow.

Across their considerable phylogenetic distance, these three case studies illustrate that meiosis as cellular core of sexuality is usually embedded in a rich functional context. The competence for sexual development in *Chara* is dependent on developmental progression and regulated by light, the access of male gametophytes to fertilisation in Maxillariinae orchids is controlled by genetic checkpoints, and fertilisation in eusocial termites is rendered independent from the presence of males by complex secretory activity in spermatheca and colleterial glands. As different as these examples are, they illustrate the degree of control these different life forms exert on their sexual propagation. Since sexuality means to give up full control of the own genetic integrity, such a high degree of control is to be expected. It can be understood as manifestation of autonomy, a central feature of any living being.
